# Derivatization-Enhanced Analysis of Glucocorticoids for Structural Characterization by Gas Chromatography-Orbitrap High-Resolution Mass Spectrometry

**DOI:** 10.3390/molecules29010200

**Published:** 2023-12-29

**Authors:** Yuqi Ge, Mengpan Liu, Xiaojun Deng, Lei Liao

**Affiliations:** Shanghai Anti-Doping Laboratory, Shanghai University of Sport, 399 Changhai Road, Shanghai 200438, China; geyuqi@sus.edu.cn (Y.G.); liumengpan@sus.edu.cn (M.L.);

**Keywords:** glucocorticoids, structural characterization, enhanced derivatization, GC-MS, high resolution

## Abstract

Glucocorticoids are classified in section S9 of the Prohibited List of the World Anti-Doping Agency, due to a potential risk to improving physical performance and causing harm to the health of athletes. Based on the similar physiological actions of glucocorticoids, both differentiating known glucocorticoids and identifying unknown glucocorticoids are important for doping control. Gas chromatography coupled with mass spectrometry plays an important role in structural characterization because of abundant structural diagnostic ions produced by electron ionization. It also provides a chance to study the fragmentation patterns. Thus, an enhanced derivatization procedure was optimized to produce trimethylsilylated glucocorticoids and structural diagnostic ions of nineteen trimethylsilylated glucocorticoids were obtained by gas chromatography-orbitrap high-resolution mass spectrometry. In our study, glucocorticoids were classified as: 3-keto-4-ene, 1,4-diene-3-keto, 3α-hydroxy with saturated A-ring, 21-hydroxy-20-keto and halo substituent glucocorticoids based on their structural difference. Structural diagnostic ions that contributed to structural characterization were specifically presented and the fragment patterns were demonstrated according to the above categories. This study not only gave new insights into the structural characterization of these glucocorticoids but also provided evidence for tracing unknown glucocorticoids or chemically modified molecules.

## 1. Introduction

Glucocorticoids have been widely used in the treatment of inflammatory and immunological diseases, while the anti-inflammatory effects make them the choice for treating asthma and painful chronic musculoskeletal injuries [1,2,3,4,5]. However, scientific evidence indicates that glucocorticoids can improve physical performance after systemic administration and cause harm to the health of athletes. Because a total glucocorticoid exposure to the body is much greater than the highest level of normal physiological cortisol production and may cause undesirable effects, such as insulin inhibition [6], muscle wasting and weakness [7,8,9], and osteoporosis [10]. Moreover, glucocorticoids could significantly inhibit the production of cortisol, aldosterone, and dehydroepiandrosterone (DHEA) by negative feedback of the hypothalamic-pituitary-adrenal (HPA) axis [11,12,13]. Glucocorticoids are therefore forbidden by the World Anti-Doping Agency (WADA) in In-Competition when glucocorticoids are administered by any injectable, oral [including oromucosal (e.g., buccal, gingival, sublingual)] or rectal route [14,15,16]. Concerning therapeutic purposes, athletes who administrate glucocorticoids should apply for a Therapeutic Use Exemption (TUE) or follow the minimum washout periods, expressed from the time of administration to the start of the In-Competition period [17]. In particular, only a handful of representative glucocorticoids are presented in the Prohibited List issued by WADA, while other substances with similar molecular structure or similar biological effects are also covered by the comment “Including but not limited to” [16]. The open feature of the Prohibited List allows any new substance to be automatically prohibited without waiting for further scientific research if the new substance is similar to those representative examples in molecular structure or biological effects. Meanwhile, an open list means not only differentiating representative glucocorticoids but also identifying unknown glucocorticoids is important for doping control.

**Figure 1:**
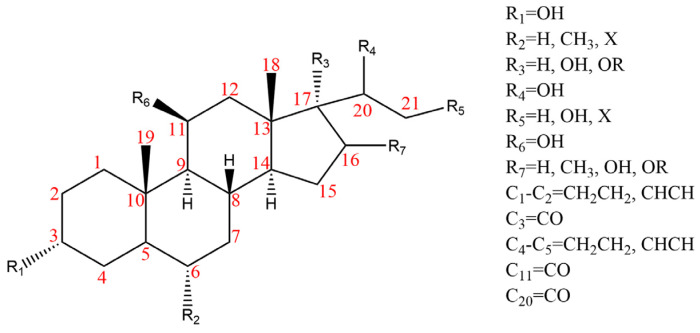
The base skeleton of glucocorticoids.

Due to the polarity and boiling point, liquid chromatography supersedes gas chromatography and becomes a widely used technique for monitoring glucocorticoids abuse in a biological matrix (e.g., urine, serum, and blood) [18,19,20,21,22]. Even though the combination of liquid chromatography and mass spectrometry provides a good sensitivity for detection, the structural diagnostic ions are still insufficient. Although the patent molecular structure is similar for all the glucocorticoids, the modifications of A-ring and D-ring differentiate certain glucocorticoids from others (Figure 1). The physico-chemical properties, pharmaco-toxicological properties, and biological activity are changed at the same time. Generally, glucocorticoids contain a side chain at the C_17_ position. An additional double bond at the C_1_ and C_2_ positions is characteristic of synthetic analogs of endogenous glucocorticoids. The substitution of one or more hydro atoms for halogen atoms often provides positive alterations to its biological activity. The lack of structural diagnostic ions means deficiencies in structural characterization and fragmentation patterns demonstration. It probably leads to the missing of unknown substances in routine analysis. Considering the importance of monitoring glucocorticoids abuse, gas chromatography combined mass spectrometry which has the advantages of obtaining sufficient structural diagnostic ions, demonstrating fragment patterns, and characterizing structure, should be paid more attention in routine analysis.

Contrary to the soft ionization technique, electron ionization (EI) could produce abundant structural diagnostic ions [23]. Furthermore, the ionization mechanism of EI is more predictable than electrospray ionization (ESI) and atmospheric pressure chemical ionization (APCI) [24]. The novel Orbitrap mass analyzer that can operate in high-resolution full scan acquisition mode with mass accuracy lower than 2 ppm makes the structural characterization more precise. Based on these features, gas chromatography coupled with orbitrap high-resolution mass spectrometry with electron ionization occupies an important position in structural characterization [25,26,27,28].

The aim of this study was to demonstrate the general laws of fragment patterns and structural characterization of glucocorticoids. For this objective, gas chromatography coupled with orbitrap high-resolution mass spectrometry was the preferred choice. Considering the difficulties in directly detecting glucocorticoids by gas chromatography coupled to mass spectrometry, a derivatization procedure is needed to decrease the boiling points of glucocorticoids and enhance the gas chromatography performance of glucocorticoids. Thus, the derivatization-enhanced procedure was optimized by comparing the chromatographic intensities of cortisone, cortisol, tetrahydrocortisone, tetrahydrocortisol, prednisone, and prednisolone. During the optimization of the derivatization procedure, the chromatographic intensities of parent ions with *m/z* at 576.31171, 650.36688, 652.38253, 726.43771, 574.29606, and 648.35123 were monitored. After being optimized, the derivatization-enhanced procedure combined with gas chromatography-orbitrap high-resolution mass spectrometry was applied to analyze nineteen glucocorticoids (Table 1). The obtained structural diagnostic ions were used to characterize glucocorticoid structures and demonstrate fragmentation patterns. It was worth noticing that several structural diagnostic ions were characteristic and could be applied to characterize the structure of glucocorticoids. Based on that, the special fragment patterns that were not reported in previous studies were demonstrated for the first time. For doping control, not only the characteristic structural diagnostic ions but also the special fragment patterns would contribute to differentiating representative glucocorticoids and identifying unknown glucocorticoids.

## 2. Results

### 2.1. Optimization of the Derivatization Procedure

#### 2.1.1. Optimization of the Derivatization Reagent

Since MSTFA was synthesized by Professor Manfred Donike [29], it has been widely used for the selective derivatization of hydroxyl. After a mild and stable derivatization procedure, the synthesized trimethylsilylated hydroxyls decrease the boiling point of targets (e.g., androgens, estrogens, and progestogens) and enhance the gas chromatography performance of targets. Due to these advantages of MSTFA, it has been applied in various different fields for over half a century, especially in anti-doping analysis [26,30,31,32]. However, the structure of glucocorticoids and androgens, estrogens, or progestogens are different, especially at the C_17_ and C_11_ positions (Table 1). Both the side chain on C_17_ and the hydroxy or keto residues on C_11_ can decrease the efficiency of derivatization [33]. Moreover, the mild characteristic of MSTFA prevents glucocorticoids from synthesizing enough trimethylsilylated hydroxyls. Thus, a more aggressive derivatization reagent is needed to improve the derivatization efficiency and the gas chromatography performance of glucocorticoids.

**Figure 2:**
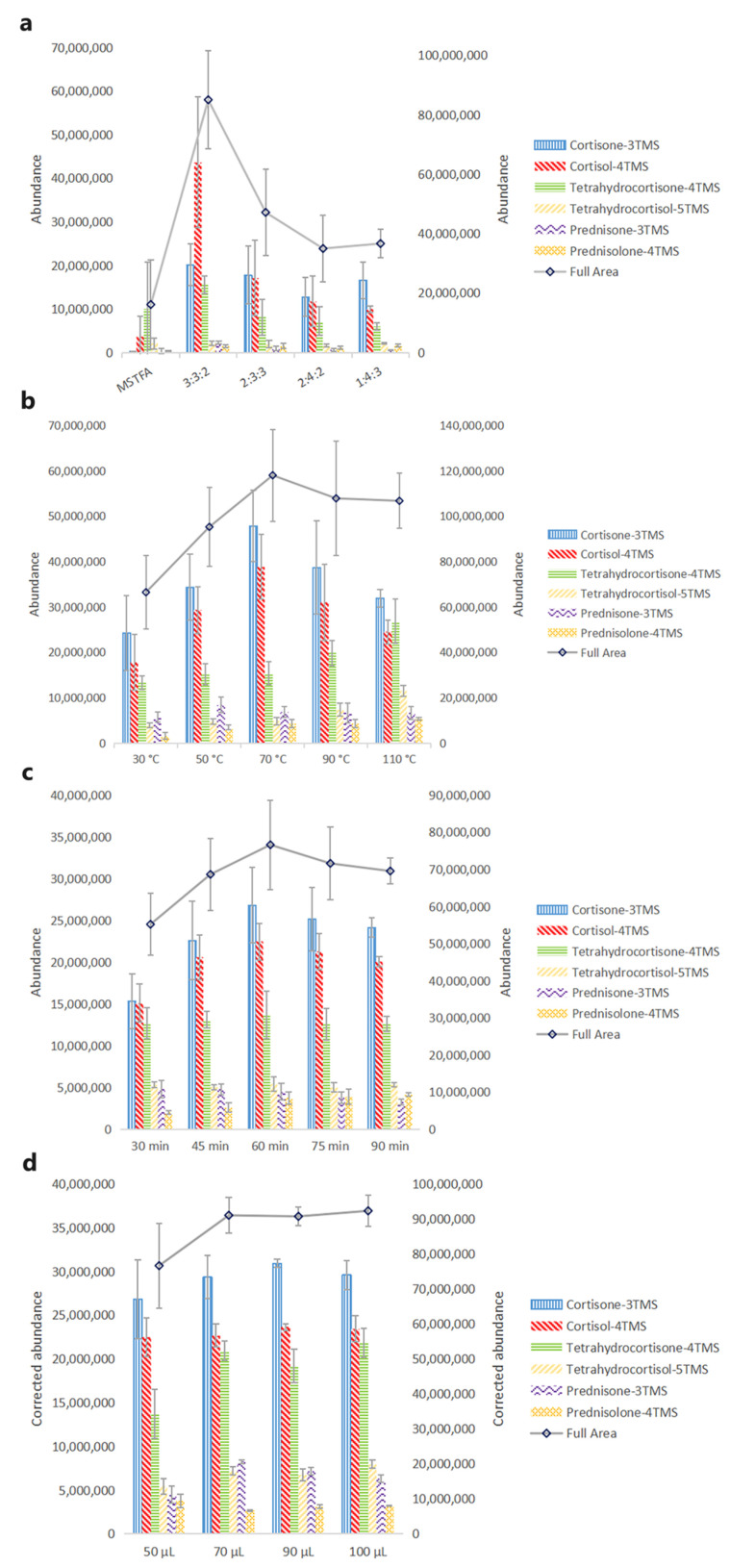
Effect of variables on chromatographic intensities. (**a**) Derivatization reagents-MSTFA vs. different mixtures of TSIM, BSA, and TMCS (*v*/*v*/*v*): concentrations: 4 μg·mL^−1^; derivatization temperature: 70 °C; derivatization duration: 30 min; derivatization reagent volume: 50 μL; (**b**) derivatization temperature: concentrations: 4 μg·mL^−1^; derivatization reagent: a 3:3:2 (*v*/*v*/*v*) mixture of TSIM, BSA, and TMCS; derivatization duration: 30 min; derivatization reagent volume: 50 μL; (**c**) derivatization duration: concentrations: 4 μg·mL^−1^; derivatization reagent: a 3:3:2 (*v/v/v*) mixture of TSIM, BSA, and TMCS; derivatization temperature: 70 °C; derivatization reagent volume: 50 μL; (**d**) derivatization reagent volume: concentrations: 4 μg·mL^−1^; derivatization reagent: a 3:3:2 (*v/v/v*) mixture of TSIM, BSA, and TMCS; derivatization temperature: 70 °C; derivatization duration: 60 min. Deviations accessed from three replications.

Several derivatization reagents have been reported in previous work, such as *N*-trimethylsilylimidazole (TSIM), *N,O*-bis-(trimethylsilyl)acetamide (BSA), trimethylchlorosilane (TMCS), trimethyliodosilane (TMSI) and so on [33]. R. P. Evershed *et al.* derivatized ecdysteroids which contain a side chain on C_17_ by TSIM [34] and J. Girault *et al.* derivatized dexamethasone by TSIM [35]. TSIM has been proven to be the most aggressive derivatization reagent on hydroxyl groups, while it does not react with amino groups or carbonyl groups. For most glucocorticoids, it still needs a derivatization reagent that could derivatize carbonyl groups. P. M. Sinpson and L. Amendola *et al.* added BSA into TSIM to improve the derivatization efficiency of glucocorticoids [36,37]. Furthermore, TMCS was also added into derivatization reagents considering its catalytic activity and its application in commercial derivatization reagents. Based on the literature review, different ratios of TSIM, BSA, and TMCS were initially tested. For cortisone, cortisol, tetrahydrocortisone, tetrahydrocortisol, prednisone, and prednisolone, the mixture of TSIM, BSA, and TMCS provided a more significant increase than MSTFA in chromatographic intensity (Figure 2a). The highest abundance of cortisone, cortisol, tetrahydrocortisone, and prednisone was at a 3:3:2 (*v*/*v*/*v*) mixture of TSIM, BSA, and TMCS, while the highest abundance of tetrahydrocortisol and prednisolone was at a 1:4:3 (*v*/*v*/*v*) mixture of TSIM, BSA, and TMCS. Considering the full area of six glucocorticoids, a 3:3:2 (*v*/*v*/*v*) mixture of TSIM, BSA, and TMCS showed better chromatographic intensity. Thus, a 3:3:2 (*v*/*v*/*v*) mixture of TSIM, BSA, and TMCS was selected for further experiments.

#### 2.1.2. Optimization of Derivatization Temperature

Derivatization is a chemical reaction that needs energy to trigger it. In previous studies, target compounds were usually derivatized with MSTFA at a high temperature for the designed time. In our study, 30 °C, 50 °C, 70 °C, 90 °C, and 110 °C were taken into consideration. As shown in Figure 2b, a significant increase in chromatographic intensities was observed with an increase in temperature; 70 °C was suitable for cortisone and cortisol, and 50 °C was suitable for prednisone. For the other glucocorticoids, 110 °C was more beneficial. The full area of six glucocorticoids increased and leveled out after the derivatization temperature was over 70 °C. Therefore, 70 °C was selected for further experiments.

#### 2.1.3. Optimization of Derivatization Duration

Except for the high temperature, a sufficient duration of derivatization also contributes to improving the derivatization efficiency. For the full area, Figure 2c clearly showed that the chromatographic intensities increased with the increase of derivatization duration, and peaked at 60 min. Cortisone and cortisol showed the same phenomenon, and no significant improvement was observed in other glucocorticoids. Therefore, 60 min was selected for further experiments.

#### 2.1.4. Optimization of Derivatization Reagent Volume

The volume of the derivatization reagent is another factor that might affect the derivatization efficiency. A small volume would result in insufficient derivatization, while a large volume would dilute the chromatographic intensity and decrease the sensitivity. So, 50 μL, 70 μL, 90 μL, and 100 μL were evaluated to acquire a sufficient volume of derivatization reagent. In order to acquire a precise result and avoid the effect of dilution, the chromatographic intensities of glucocorticoids were corrected by the dilution factor (e.g., abundance × volume × 50^−1^). For most glucocorticoids, the increase of chromatographic intensity reached a plateau after the volume increased to 70 μL (Figure 2d). Thus, 70 μL was sufficient in most conditions.

### 2.2. Structural Characterization of Glucocorticoids

**Figure 3:**
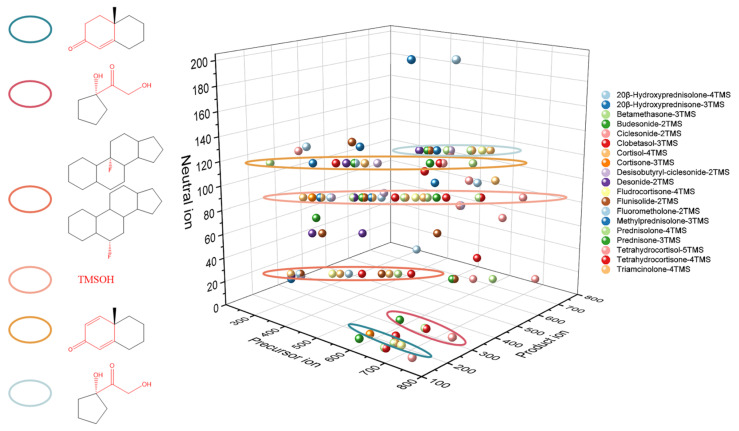
Structural diagnostic ions of nineteen glucocorticoids.

An ideal derivatization procedure not only enhances the capacity of gas chromatography but also provides structural diagnostic ions which is the first step of structural characterization and contributes to the complete understanding of fragmentation patterns. In our study, the derivatization-enhanced method was applied to analyze nineteen glucocorticoids (Figure S1). Structural diagnostic ions observed are listed in Table 2. As shown in Figure 3, the same structural diagnostic ions indicated that there were similar fragmentation patterns in different glucocorticoids.

In order to elucidate fragmentation patterns of glucocorticoids and make the structural characterization of glucocorticoids more clear, nineteen glucocorticoids were classified into five categories based on the small differences between structures. The fragmentation patterns of glucocorticoids were discussed according to the above five categories and obtained structural diagnostic ions. It was worthwhile to point out that the observations always considered the derivatized (i.e., trimethylsilylated) structure of the target glucocorticoids. Meanwhile, the neutral loss of TMSOH (*m/z* 90) which was a common fragment in trimethylsilylated derivatization was not considered in the discussion.

#### 2.2.1. Class I: 3-Keto-4-Ene Glucocorticoids

**Figure 4:**
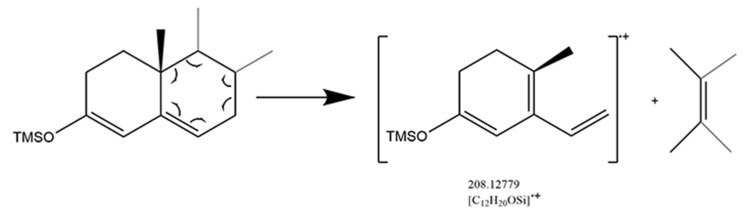
Mechanism of Retro Diels-Alder fragmentation in 3-keto-4-ene glucocorticoids.

The 3-keto-4-ene structure was dominated by Retro Diels-Alder fragmentation that resulted in the B-ring cleavage and conjugated diene radical cation produced (Figure 4). Cyclohexane with a cyclic olefinic bond is the key to Retro Diels-Alder fragmentation. The derivatization converted the 3-keto structure into a 3-enol structure, then transferred the double bond from the C_4_ position to the C_5_ position, providing a chance to perform Retro Diels-Alder fragmentation. As the mass spectrum of cortisone, cortisol, and fludrocortisone shown in Figure S2, conjugated diene radical cation ([C_12_H_20_OSi]^·+^) with *m/z* at 208.12779 was observed after the Retro Diels-Alder fragmentation. It could be selected as a structural diagnostic ion, despite the fact that it is generally of low abundance.

#### 2.2.2. Class II: 1,4-Diene-3-Keto Glucocorticoids

**Figure 5:**
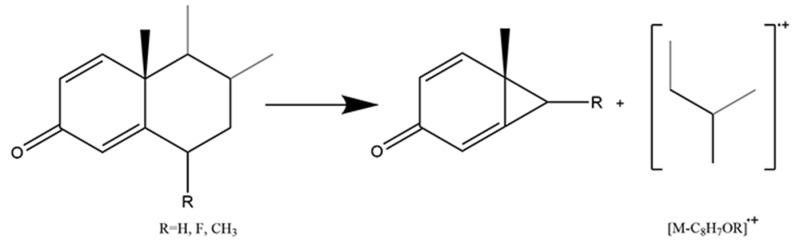
Mechanism of fission of bonds C_6_/C_7_ and C_9_/C_10_ in 1,4-diene-3-keto glucocorticoids.

Different from Class I, fourteen glucocorticoids in this class are synthetic. Based on the endogenous glucocorticoids, scientists introduced an additional double bond in the A-ring which led to A-ring flattening and increased the binding affinity for the receptor [38,39]. Moreover, the conjugated double bonds extending from C_1_ and C_3_ to C_4_ decreased the derivatization efficiency of the 3-keto residue. Instead of the Retro Diels-Alder fragmentation pattern, the B-ring cleavage occurred with the fission of bonds C_6_/C_7_ and C_9_/C_10_ (Figure 5). The neutral loss of [C_8_H_7_OR] was observed in fourteen 1,4-diene-3-keto glucocorticoids (Table 3). In addition, Retro Diels-Alder fragmentation was also performed on a few 1,4-diene-3-keto glucocorticoids, such as prednisolone. It could produce a conjugated diene radical cation ([C_12_H_18_OSi]^·+^) with *m/z* at 206.11214. They both represented typical fragmentation patterns and contributed to distinguishing 1,4-diene-3-keto glucocorticoids from others.

#### 2.2.3. Class III: 3α-Hydroxy with Saturated A-Ring Glucocorticoids

**Figure 6:**
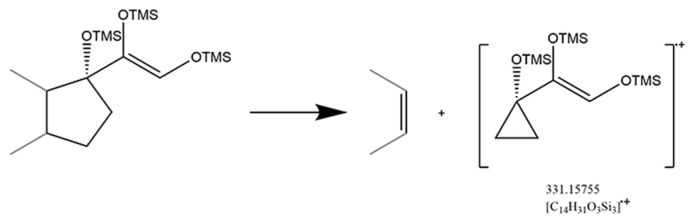
Mechanism of fission of bonds C_13_/C_17_ and C_14_/C_15_ in 3α-hydroxy with saturated A-ring glucocorticoids.

A saturated A-ring was normal in anabolic androgenic steroids, while two glucocorticoids were part of this category. Although the saturated A-ring and the reduction of 3-keto to 3α-hydroxy were not favorable for receptor binding, the structure characteristics were suitable for trimethylsilylated derivatization. The 3-keto residue would inhibit the 20-keto residue for trimethylsilylated derivatization, while the 3α-hydroxy residue could increase the derivatized efficiency of the 20-keto residue that produced a fragment pattern with fission of bonds C_13_/C_17_ and C_14_/C_15_ (Figure 6). A significant diagnostic ion ([C_14_H_31_O_3_Si_3_]^·+^) with *m/z* at 331.15755 existed after the D-ring cleavage (Figure S3). Moreover, the substantial abundance of the [M]^·+^ and [M-15]^·+^ was another feature of 3α-hydroxy with saturated A-ring glucocorticoids.

#### 2.2.4. Class: IV: 21-Hydroxy-20-Keto Glucocorticoids

**Figure 7:**
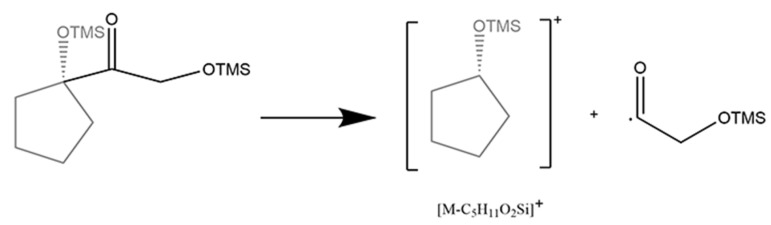
Mechanism of cleavage of side chain at C_17_ position in 21-hydroxy-20-keto glucocorticoids.

The 20-keto group and the hydroxy at the C_21_ position are the characteristic structure of glucocorticoids. Fourteen glucocorticoids belong to this class, which concerned two fragment patterns. For tetrahydrocortisol, tetrahydrocortisone, and prednisone, the cleavage of D-ring was performed as described (Figure 6). For the other glucocorticoids with an unsaturated A-ring, it could be characterized by the cleavage of the side chain at the C_17_ position (Figure 7) which leads to the fission of bond C_17_/C_20_ (Table 4). In particular, two different and competitive fragment patterns occurred in prednisolone at the same time. Both the significant diagnostic ion ([C_14_H_31_O_3_Si_3_]^·+^) with *m/z* at 331.15755 and the loss of [C_5_H_11_O_2_Si] with *m/z* at 131.05228 could imply the existence of glucocorticoids.

#### 2.2.5. Class V: Halogen Substituent Glucocorticoids

Halogen substituent at the C_9_ or C_6_ position is a common modification in glucocorticoids, which could provide higher biological activity than endogenous glucocorticoids. A series of diagnostic ions at [M]^·+^ and [M-HF]^·+^, resulting from halogen substituent cleavage, were characteristic of the presence of halogen substituent and were observed in six glucocorticoids (Table 5).

## 3. Discussion

Though liquid chromatography combined with mass spectrometry has been widely applied to analyze glucocorticoids, this derivatization-enhanced procedure combined with gas chromatography-orbitrap high-resolution mass spectrometry optimized in this study has many advantages over other analytical techniques.

Compared to the conventional LC-MS methods [18,19,20,21,22], gas chromatography-orbitrap high-resolution mass spectrometry provides sufficient structural diagnostic ions with a high mass accuracy and fills in gaps in structural characterization by chromatographic techniques. Moreover, it shows wide application prospects in differentiating representative substances and identifying unknown substances for doping control. Based on our previous work operating on a conventional S/SL injection mode, analyzing glucocorticoids by GC-MS without derivatization did not obtain satisfactory results. Though the polarity and boiling points of glucocorticoids make the analysis difficult to directly operate in GC-MS, the chromatographic intensities of glucocorticoids could be significantly improved after derivatization. Compared with previous derivatization methods, this optimized derivatization procedure not only improves the chromatography performance but also simplifies the pretreatment procedure. The reaction time was shortened to 1 h in this study, while other derivatization methods need over 10 h [36,40,41] and additional assisted equipment [37,42].

Previous studies on glucocorticoids mainly focused on improving detection limits [43,44,45], optimizing pretreatment procedures [46,47,48], and evaluating pharmacokinetics [49,50]. Using this derivatization-enhanced procedure combined with gas chromatography-orbitrap high-resolution mass spectrometry, nineteen glucocorticoids were analyzed to obtain structural diagnostic ions and reveal the fragment patterns of glucocorticoids. Several representative fragment patterns of glucocorticoids reported in this study have not been studied before. These special fragment patterns represent key structural characteristics of glucocorticoids and could be applied to monitoring glucocorticoids abuse in competitions. Additionally, the results could also be benefit for identifying new metabolites of glucocorticoids in post-administration urine with pretreatment procedures, such as hydrolysis, extraction, and enhanced derivatization.

## 4. Materials and Methods

### 4.1. Materials

All organic solvents of HPLC grade were obtained from Sigma-Aldrich (Saint Louis, MO, USA). *N*-trimethylsilylimidazole (TSIM), *N,O*-bis-(trimethylsilyl)acetamide (BSA), trimethylchlorosilane (TMCS), *N*-methyl-*N*-trifluorotrimethylsilylacetamide (MSTFA), ammonium iodide (NH_4_I) and ethanethiol which were used for derivatization were also obtained from Sigma-Aldrich.

20β-Hydroxyprednisolone, 20β-hydroxyprednisone, cortisone, fludrocortisone, flunisolide, tetrahydrocortisol, and tetrahydrocortisone were purchased from Alta (Tianjin, China). Clobetasol and desonide were purchased from Toronto Research Chemicals (Toronto, ON, Canada). Betamethasone, budesonide, ciclesonide, cortisol, desisobutyryl-ciclesonide, fluorometholone, methylprednisolone, prednisolone, prednisone, and triamcinolone were supplied by Dr. Ehrenstorfer GmbH (Augsburg, Germany).

Stock solutions of glucocorticoids were prepared in methanol at a concentration of 1 mg·mL^−1^, then were diluted to prepare working solutions with appropriate dilution factors. All solutions were stored at −20 °C until usage.

### 4.2. Derivatization

The appropriate volume of glucocorticoids was evaporated to dryness at 55 °C waiting for derivatization. The dried residue was derivatized with 70 µL of TSIM/BSA/TMCS (3:3:2, *v/v/v*), which was heated at 70 °C for 30 min previously, for 60 min at 70 °C. 2 µL of the derivatized glucocorticoids was injected in the GC-MS system for analysis.

### 4.3. GC-MS Analysis

GC-MS analysis was performed on an Orbitrap Exploris GC 240 equipped with an HP-1 column (25 m × 0.2 mm × 0.11 µm). The injector was set at 280 °C and each sample (2 μL) was injected with a split ratio of 10:1. The oven temperature was initially set at 200 °C then increased at 15 °C/min to 260 °C, then the temperature was increased at 10 °C/min to 320 °C and maintained at final temperature for 5 min. The total analysis time was 15 min. The transfer line was kept at 300 °C during analysis. Helium (99.999%) was used as carrier gas at a constant pressure of 21.500 psi.

The ion source temperature and electron energy were kept at 250 °C and 70 eV, respectively. Full scan acquisition mode was applied with a mass range of 100–750 and a high resolution of 120,000 at *m/z* 200. Qualitative and quantitative analysis was operated by structural diagnosis ions (Table 2).

### 4.4. Statistical Analysis

Thermo Fisher Xcalibur software (Version 4.4), PerkinElmer ChemDraw software (Version 19.0), and OriginLab software (Version 2019b) were utilized for data analysis.

## 5. Conclusions

In the present study, an enhanced derivatization procedure which could improve the derivatization efficiency of glucocorticoids and highlight more specific features, was optimized, involving derivatization reagents, derivatization duration, derivatization temperature, and derivatization reagent volume. With the proposed procedure, nineteen glucocorticoids were analyzed. The structural characterization centered on the mass spectrometric behavior of glucocorticoids. The loss of [C_5_H_11_O_2_Si] with *m/z* at 131.05228 indicated a side chain on C_17_. The significant structural diagnostic ion ([C_14_H_31_O_3_Si_3_]^·+^) with *m/z* at 331.15755 represented a saturated D-ring with a hydroxyl group and a side chain on C_17_. Both of them could be characteristic of glucocorticoids. Conjugated diene radical cation with *m/z* at 208.12779 ([C_12_H_20_OSi]^·+^) means the existence of 4-ene glucocorticoids, which could be produced endogenously. Conjugated diene radical cation with *m/z* at 206.11214 ([C_12_H_18_OSi]^·+^) and the neutral loss of [C_8_H_7_OR] both implied the existence of 1,4-diene glucocorticoids, which usually had an exogenous origin. A series neutral loss of HX implied the modification of glucocorticoids with heteroatoms. The obtained structural diagnostic ions contributed to demonstrating special fragmentation patterns and gave new insights into the structural characterization of these glucocorticoids. For doping control, these structural diagnostic ions and special fragmentation patterns provided a chance to differentiate known glucocorticoids. Moreover, the results of this study also suggested that the special fragmentation patterns could allow to trace novel glucocorticoids which would be invisible to the current analytical methods.

## Figures and Tables

**Table 1:** Chemical characteristics of glucocorticoids.

NO.	Compounds	CAS	Chemical Formula	Structure
1	20β-Hydroxyprednisolone	15847-24-2	C_21_H_30_O_5_	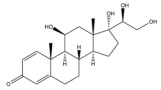
2	20β-Hydroxyprednisone	600-92-0	C_21_H_28_O_5_	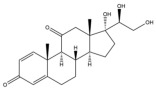
3	Betamethasone	378-44-9	C_22_H_29_FO_5_	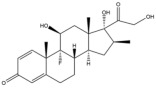
4	Budesonide	51372-29-3	C_25_H_34_O_6_	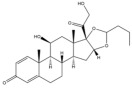
5	Ciclesonide	126544-47-6	C_32_H_44_O_7_	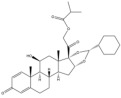
6	Clobetasol	25122-41-2	C_22_H_28_ClFO_4_	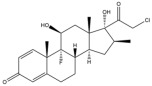
7	Cortisol	50-23-7	C_21_H_30_O_5_	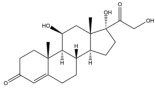
8	Cortisone	53-06-5	C_21_H_28_O_5_	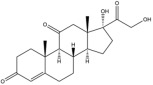
9	Desisobutyryl-ciclesonide	161115-59-9	C_28_H_38_O_6_	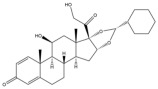
10	Desonide	638-94-8	C_24_H_32_O_6_	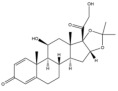
11	Fludrocortisone	127-31-1	C_21_H_29_FO_5_	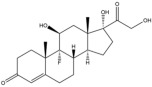
12	Flunisolide	3385-03-3	C_24_H_31_FO_6_	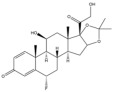
13	Fluorometholone	426-13-1	C_22_H_29_FO_4_	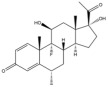
14	Methylprednisolone	83-43-2	C_22_H_30_O_5_	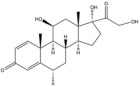
15	Prednisolone	50-24-8	C_21_H_28_O_5_	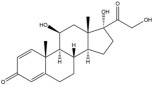
16	Prednisone	53-03-2	C_21_H_26_O_5_	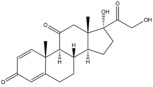
17	Tetrahydrocortisol	53-02-1	C_21_H_34_O_5_	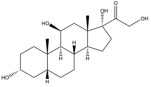
18	Tetrahydrocortisone	53-05-4	C_21_H_32_O_5_	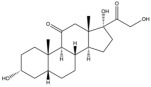
19	Triamcinolone	124-94-7	C_21_H_27_FO_6_	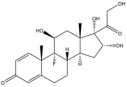

**Table 2:** Structural diagnostic ions of nineteen glucocorticoids in the present study.

NO.	Derivatized Compounds	Diagnostic Ions ^a^	Structural Categories ^b^
1	20β-Hydroxyprednisolone-4TMS	650.36688, 547.30896, 445.25887, 355.20878, 325.20136, 265.15887	Class II
2	20β-Hydroxyprednisone-3TMS	576.31170, 473.25378, 371.20369, 281.25361, 251.14618	Class II
3	Betamethasone-3TMS	608.31793, 477.26510, 457.25887, 387.21501, 367.20878, 297.16492, 277.15869, 177.10741	Class II, Class IV, Class V
4	Budesonide-2TMS	574.31404, 443.26121, 353.21112, 323.20370, 281.15361	Class II, Class IV
5	Ciclesonide-2TMS	684.38721, 612.34768, 529.26161, 483.29251, 477.26669, 389.21426, 299.16417, 281.15361, 263.14304	Class II
6	Clobetasol-3TMS	626.28404, 591.31519, 501.26510, 481.25887, 471.25767, 411.21501, 391.20878, 291.09978, 243.12311	Class II, Class V
7	Cortisol-4TMS	650.36688 *, 519.31405, 447.27452, 429.26396, 357.22443, 339.21386, 267.17434, 249.16378, 208.12779	Class I, Class IV
8	Cortisone-3TMS	576.31171 *, 445.25887, 355.20878, 265.15869, 208.12779	Class I, Class IV
9	Desisobutyryl-ciclesonide-2TMS	614.34534, 531.25927, 483.29251, 389.21426, 363.23500, 299.16417, 281.15361, 263.14304	Class II, Class IV
10	Desonide-2TMS	560.29839, 429.24556, 371.20370, 339.19547, 309.18805, 281.15361, 263.14304	Class II, Class IV
11	Fludrocortisone-4TMS	668.35746, 537.30462, 447.25454, 427.24831, 357.20445, 337.19822, 208.12779	Class I, Class IV, Class V
12	Flunisolide-2TMS	578.28897, 505.22363, 447.23614, 427.22991, 357.18605, 309.18805, 299.14418, 279.13796	Class II, Class IV, Class V
13	Fluorometholone-2TMS	520.28349, 477.26510, 387.21501, 367.20878, 297.16492, 277.15869, 233.13562	Class II, Class V
14	Methylprednisolone-3TMS	590.32736, 459.27452, 369.22443, 325.20136, 279.17434, 264.15087	Class II, Class IV
15	Prednisolone-4TMS	648.35123, 633.32776, 558.30114, 528.29372, 468.25105, 331.15755, 169.06793	Class II, Class IV
		648.35123 *, 558.30114, 517.29840, 427.24831, 337.19822, 206.11214	Class II, Class IV
16	Prednisone-3TMS	574.29606 *, 559.27258, 484.25496, 454.23854, 331.15755, 169.06793	Class II, Class IV
17	Tetrahydrocortisol-5TMS	726.43771 *, 711.41423, 636.38762, 531.31405, 331.15755, 169.06793	Class III, Class IV
18	Tetrahydrocortisone-4TMS	652.38253 *, 562.33244, 449.29017, 331.15755, 169.06793	Class III, Class IV
19	Triamcinolone-4TMS	682.33673, 577.26316, 551.28389, 461.23380, 441.22757, 371.18371, 351.17748, 341.17629, 281.13362, 261.12739	Class II, Class IV, Class V

^a^. The diagnostic ions listed above with * markings were quantitative ions, which were monitored during the optimization of the derivatization procedure. ^b^. Class I: 3-keto-4-ene glucocorticoids; Class II: 1,4-diene-3-keto glucocorticoids; Class III: 3α-hydroxy with saturated A-ring glucocorticoids; Class IV: 21-hydroxy-20-keto glucocorticoids; Class V: halogen substituent glucocorticoids.

**Table 3:** Neutral loss found in 1,4-diene-3-keto glucocorticoids.

NO.	Derivatized Compounds	Diagnostic Ions	Fragmentation Patterns
1	20β-Hydroxyprednisolone-4TMS	325.20136	[M-C_8_H_21_O_2_Si_2_-C_8_H_8_O]^·+^
2	20β-Hydroxyprednisone-3TMS	251.14618	[M-C_8_H_21_O_2_Si_2_-C_8_H_8_O]^·+^
3	Betamethasone-3TMS	177.10741	[M-C_11_H_31_O_4_Si_3_-C_8_H_8_O]^·+^
4	Budesonide-2TMS	323.20370	[M-C_5_H_11_O_2_Si-C_8_H_8_O]^·+^
5	Ciclesonide-2TMS	477.26669	[M-CH_3_-C_8_H_8_O]^·+^
6	Clobetasol-3TMS	471.25767	[M-Cl-C_8_H_8_O]^·+^
7	Desisobutyryl-ciclesonide-2TMS	363.23500	[M-C_5_H_11_O_2_Si-C_8_H_8_O]^·+^
8	Desonide-2TMS	309.18805	[M-C_5_H_11_O_2_Si-C_8_H_8_O]^·+^
9	Flunisolide-2TMS	309.18805	[M-C_5_H_11_O_2_Si-C_8_H_7_OF]^·+^
10	Fluorometholone-2TMS	233.13562	[M-C_5_H_13_O_2_Si-HF-C_9_H_10_O]^·+^
11	Methylprednisolone-3TMS	325.20136	[M-C_5_H_11_O_2_Si-C_9_H_10_O]^·+^
12	Prednisolone-4TMS	528.29372	[M-C_8_H_8_O]^·+^
		206.11214	[C_12_H_18_OSi]^·+^
13	Prednisone-3TMS	454.23854	[M-C_8_H_8_O]^·+^
14	Triamcinolone-4TMS	341.17629	[M-C_8_H_21_O_3_Si_2_-C_8_H_8_O]^·+^

**Table 4:** Fragmentation pattern of 21-hydroxy-20-keto glucocorticoids.

NO.	Derivatized Compounds	Diagnostic Ions	Fragmentation Patterns
1	Betamethasone-3TMS	477.26510	[M-C_5_H_11_O_2_Si]^+^
2	Budesonide-2TMS	443.26121	[M-C_5_H_11_O_2_Si]^+^
3	Cortisol-4TMS	519.31405	[M-C_5_H_11_O_2_Si]^+^
4	Cortisone-3TMS	445.25887	[M-C_5_H_11_O_2_Si]^+^
5	Desisobutyryl-ciclesonide-2TMS	483.29251	[M-C_5_H_11_O_2_Si]^+^
6	Desonide-2TMS	429.24556	[M-C_5_H_11_O_2_Si]^+^
7	Fludrocortisone-4TMS	537.30462	[M-C_5_H_11_O_2_Si]^+^
8	Flunisolide-2TMS	447.23614	[M-C_5_H_11_O_2_Si]^+^
9	Methylprednisolone-3TMS	459.27452	[M-C_5_H_11_O_2_Si]^+^
10	Prednisolone-4TMS	331.15755	[C_14_H_31_O_3_Si_3_]^·+^
		517.29840	[M-C_5_H_11_O_2_Si]^+^
11	Prednisone-3TMS	331.15755	[C_14_H_31_O_3_Si_3_]^·+^
12	Tetrahydrocortisol-5TMS	331.15755	[C_14_H_31_O_3_Si_3_]^·+^
13	Tetrahydrocortisone-4TMS	331.15755	[C_14_H_31_O_3_Si_3_]^·+^
14	Triamcinolone-4TMS	551.28389	[M-C_5_H_11_O_2_Si]^+^

**Table 5:** Fragmentation pattern of halogen substituent glucocorticoids.

NO.	Derivatized Compounds	Diagnostic Ions	Fragmentation Patterns
1	Betamethasone-3TMS	477.26510-457.25887, 387.21501-367.20878, 297.16492-277.15869	[M-HF]^·+^
2	Clobetasol-3TMS	501.26510-481.25887, 411.21501, 391.20878	
3	Fludrocortisone-4TMS	447.25454-427.24831, 357.20445-337.19822	
4	Flunisolide-2TMS	447.23614-427.22991, 299.14418-279.13796	
5	Fluorometholone-2TMS	387.21501-367.20878, 297.16492-277.15869	
6	Triamcinolone-4TMS	461.23380-441.22757, 371.18371-351.17748, 281.13362-261.12739	

## Data Availability

Data are contained within the article and Supplementary Materials.

## Supplementary Materials

The following supporting information can be downloaded at: https://www.mdpi.com/article/10.3390/molecules29010200/s1, Figure S1: The total ion chromatogram of nineteen glucocorticoids; Figure S2: Mass spectrum of cortisone (a), cortisol (b) and fludrocortisone (c); Figure S3: Mass spectrum of tetrahydrocortisol (a), tetrahydrocortisone (b) and prednisolone (c).
