# Supplementary material for: Derivatization-Enhanced Analysis of Glucocorticoids for Structural Characterization by Gas Chromatography-Orbitrap High-Resolution Mass Spectrometry
Source: Molecules. 2023 Dec 29;29(1):200. doi: 10.3390/molecules29010200 (PMC10780989; doi:10.3390/molecules29010200)
Supplement: Supplementary file 1 [file molecules-29-00200-s001.zip › molecules-2700389-supplementary.pdf]

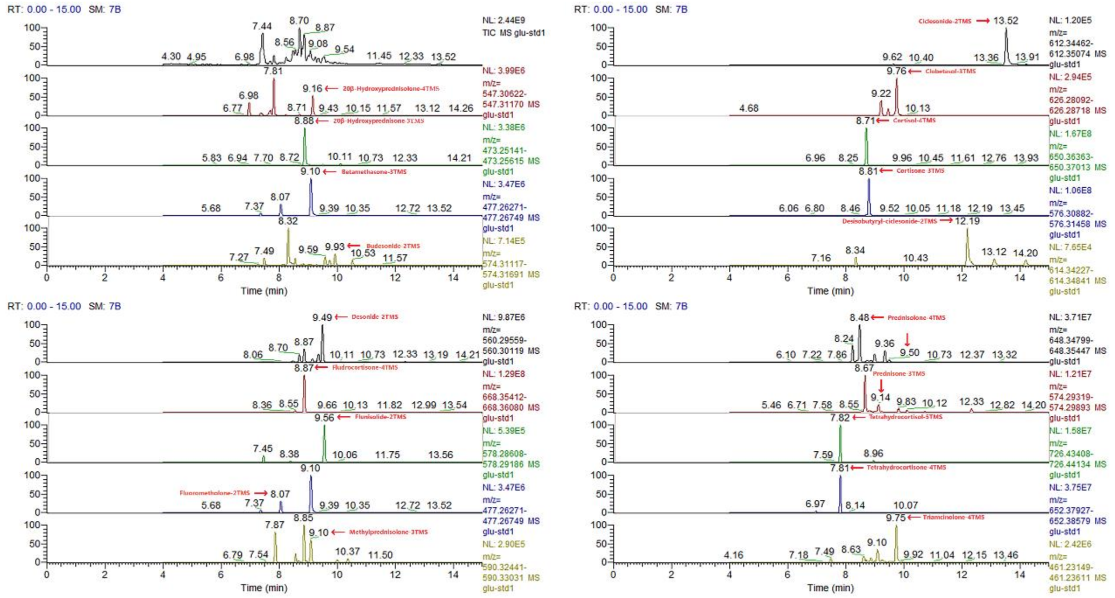

Figure S1: The total ion chromatogram of nineteen glucocorticoids.

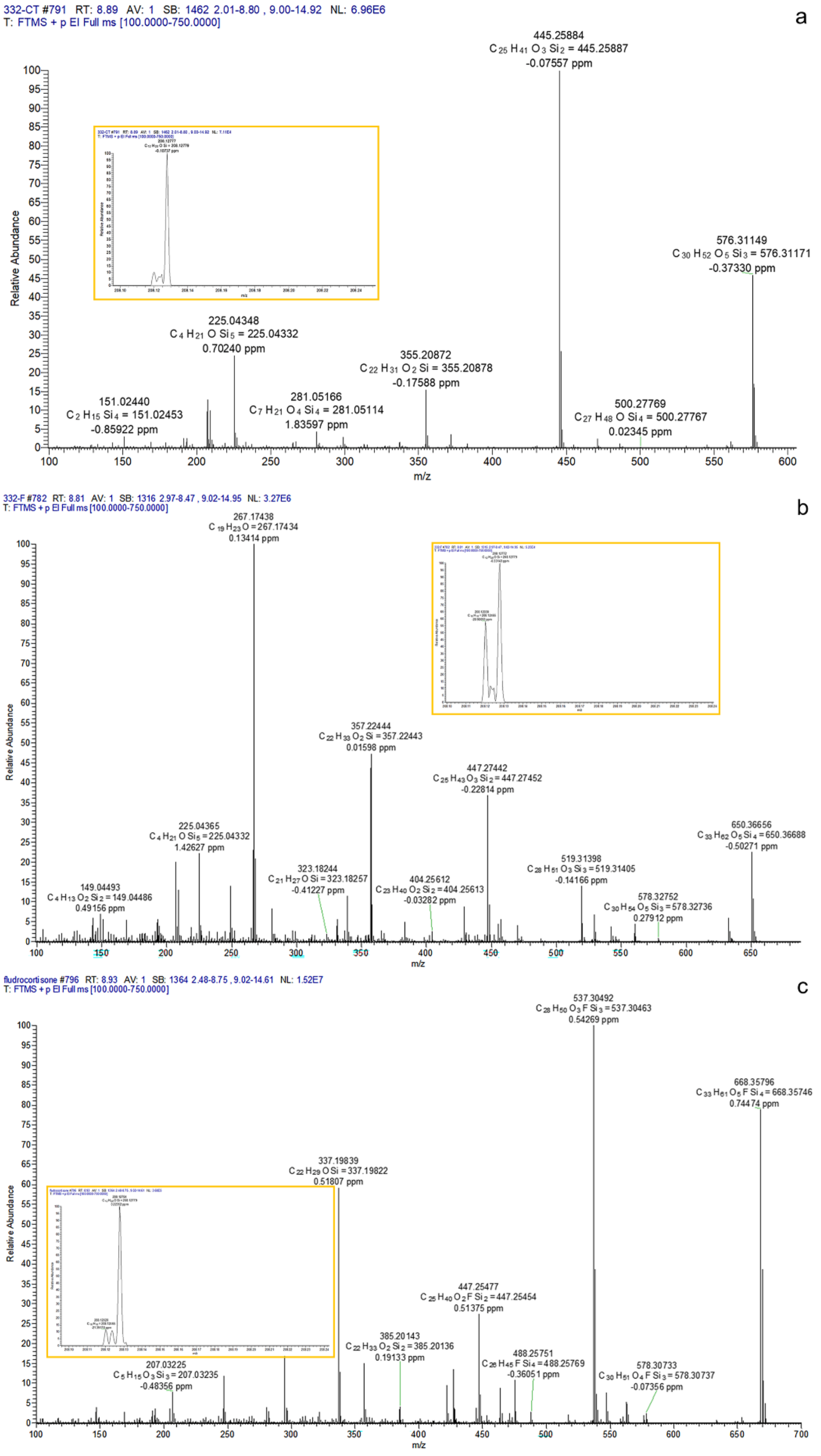

Figure S2: Mass spectrum of cortisone (**a**), cortisol (**b**) and fludrocortisone (**c**).

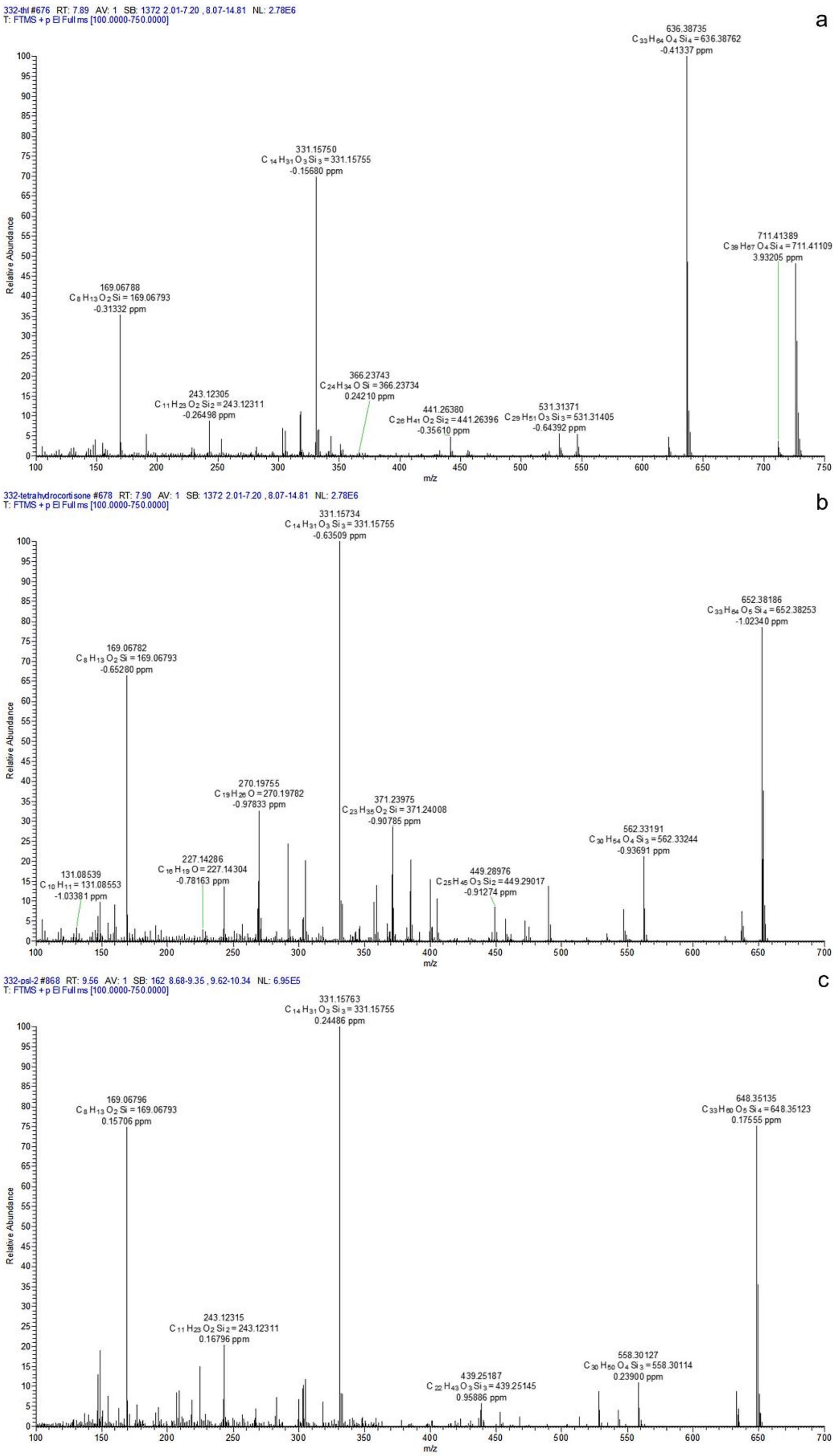

Figure S3: Mass spectrum of tetrahydrocortisol (**a**), tetrahydrocortisone (**b**) and prednisolone (**c**).
